# Perioperative NSAID use and postoperative outcomes after distal radius fracture surgery: a propensity-matched cohort study

**DOI:** 10.1007/s00590-026-04980-y

**Published:** 2026-09-30

**Authors:** Arjun Dinesh, Sandra Fahmy, Mohammad Poursalehian, Youssra Marjoua, Cesar Cereijo, Damien Billow, Ronald Peirish, Anokha Padubidri, Joseph Styron, Heather Vallier

**Affiliations:** 1 School of Medicine, Case Western Reserve University, Cleveland, USA; 2 Department of Orthopaedic Surgery, Cleveland Clinic, Cleveland, USA

**Keywords:** Distal radius fracture, NSAIDs, Postoperative complications, Propensity score matching, Nonunion

## Abstract

**Purpose:**

Nonsteroidal anti-inflammatory drugs (NSAIDs) are commonly used for pain management following distal radius fracture (DRF) surgery, yet their effects on postoperative complications remain unclear. This study evaluated the association between perioperative prescriptive NSAID use and surgical and medical complications following DRF surgery.

**Methods:**

A retrospective cohort study using the TriNetX database identified adult patients who underwent DRF surgery between 2012 and 2024. Patients were categorized based on NSAID prescription within 1 day before to 1 month after surgery. Propensity score matching controlled for demographic variables. Primary outcome was 1-year surgical complications (malunion/nonunion surgery, postoperative infection, and revision surgery). Secondary outcomes included 90-day medical complications (myocardial infarction, gastrointestinal bleeding, acute kidney injury, emergency department visits, and readmission). Statistical analyses included Kaplan-Meier survival analysis and Cox proportional hazards regression.

**Results:**

Among 76,913 patients identified, 30,760 matched pairs were analyzed. The primary outcome of malunion/nonunion surgery at 1 year showed no significant difference after matching. At 1 year, NSAID prescription patients had significantly higher rates of postoperative infection and revision surgery. Within 90 days, NSAID prescription patients demonstrated higher emergency department visit rates but no significant differences in myocardial infarction, gastrointestinal bleeding, acute kidney injury, or readmission.

**Conclusions:**

Despite statistically significant increases in postoperative infections, revision surgery, and emergency department visits, absolute risk differences were modest (less than 1% for all outcomes). No significant difference was observed in the primary outcome of malunion/nonunion surgery, and this null finding persisted across sensitivity analyses restricted to nonselective NSAIDs and to a 1-week exposure window. Given the small absolute risk differences observed and the effectiveness of NSAIDs for pain control, these findings suggest that perioperative NSAID use is not associated with a clinically meaningful increase in fracture nonunion, though the modest increases in infection and revision surgery warrant individualized risk-benefit discussion, particularly in patients with additional risk factors for these complications.

**Level of evidence:**

III.

**Supplementary Information:**

The online version contains supplementary material available at https://doi.org/10.1007/s00590-026-04980-y.

## Introduction

Effective pain management after orthopaedic surgery is essential to maximize patient safety, recovery, and outcomes [1, 2]. Nonsteroidal anti-inflammatory drugs (NSAIDs) are commonly used to manage pain following orthopedic surgeries, especially as a part of multimodal pain strategies [3]. NSAIDs exert their effects by inhibiting cyclooxygenase (COX) enzyme isoforms (COX-1 and COX-2) or selectively inhibiting COX-2 [4].

Preclinical studies have also demonstrated that non-selective NSAIDs and COX-2 inhibitors impair fracture healing [5–8]. Human studies, however, remain inconclusive and variable regarding the clinical effects of NSAIDs on nonunion rates and bone healing [9–14]. Distal radius fractures (DRFs), the most common fractures in the United States, represent a substantial proportion of cases in which postoperative NSAID is routinely used [15]. Additionally, few studies have analyzed the association between perioperative NSAID use and DRF healing [16–23].

Overall, there is a lack of comprehensive evaluation of the risks associated with perioperative use of NSAIDs following DRF surgery. The purpose of this study is to assess the risk of non-union and post-operative adverse events in patients who received nonselective NSAID prescriptions following surgery for a DRF.

## Methods

A retrospective analysis of patients presenting to US health care organizations within the TriNetX Research Network between 2012 and 2024 with operatively treated DRFs was conducted. The TriNetX Research Network is a global health-collaborative clinical research platform comprising deidentified electronic health records from 58 health care organizations across the United States with over 100 million patients. The TriNetX server extracts clinical variables directly from clinical documents using an integrated Natural Language Processing system. TriNetX ensures the protection of patient health information by providing only aggregate counts and statistical summaries, maintaining deidentification at all stages of data retrieval and dissemination.

### Eligibility

Patients aged 18 years and older who underwent surgery for DRFs, identified using ICD-10 and Current Procedure Terminology (CPT) codes, were included in the study (see Supplementary Table 1). From this population, subgroups were formed based on NSAID prescription status. The NSAID prescription cohort included patients who received any NSAID prescription within 1 day before to 1 month after the index surgical procedure. Index date was first CPT code for DRF surgery in the time window. The non-NSAID prescription cohort included patients who underwent DRF fixation but did not have a NSAID prescription within this time window. Patient demographic information, medical comorbidities, and outcomes were collected.

### Outcomes

Surgical complications, including postoperative infection, malunion/nonunion surgery, and revision surgery, were assessed for 1 year after the index surgery. Medical complications, including myocardial infarction (MI), gastrointestinal bleeding (GIB), acute kidney injury (AKI), emergency department (ED) visits, and inpatient readmission, were assessed for a 90-day period.

### Statistical analysis

Categorical data are reported as counts with percentages, while continuous data are presented as means with standard deviations. Propensity score matching (PSM) was conducted in the TriNetX platform using 1:1 nearest neighbor matching without replacement, based on a logistic regression model incorporating covariates including race (White, Black or African American), ethnicity (Hispanic or Latino), body mass index categories, opioid analgesic use, bisphosphonate use, glucocorticoid use, and comorbidities including hyperlipidemia, osteoporosis (with and without pathological fracture), diabetes mellitus, anemia, chronic obstructive pulmonary disease, chronic kidney disease, heart failure, alcohol dependence, nicotine dependence, vitamin D deficiency, rheumatoid arthritis, and malnutrition within 1 year of the index event.

An unmatched comparison was performed for the primary outcome of malunion/nonunion surgery to assess the effect size prior to covariate adjustment. After PSM, odds ratios with 95% confidence intervals (CI) were calculated to compare outcomes between cohorts. Variables with missing data are handled by the TriNetX platform through suppression of incomplete fields; no manual imputation was performed. Kaplan-Meier survival analysis was performed for time-to-event outcomes, with log-rank tests used to compare survival curves between cohorts. Hazard ratios with 95% CIs were calculated using Cox proportional hazards regression. Statistical significance was defined as *P* < 0.05. Statistical analyses were performed in the TriNetX research environment. To assess whether exposure heterogeneity influenced the observed associations, two sensitivity analyses were performed. First, patients who received a COX-2 selective inhibitor prescription were excluded from the NSAID cohort, restricting the exposure definition to nonselective NSAIDs only. Second, the NSAID exposure window was narrowed to prescriptions occurring from 1 day before to 1 week after the index surgical procedure. Both sensitivity cohorts were re-matched 1:1 to the non-NSAID cohort using the same propensity score model and covariates described above, and outcomes were assessed using the same methods.

## Results

### Demographics and comorbidities

In total, 76,913 patients with operatively treated DRFs were identified between 2012 and 2024. Of these, 32,037 patients (41.6%) received NSAID prescriptions within the predefined postoperative exposure window, whereas 44,876 patients (58.4%) did not receive NSAID prescriptions. Before matching, the mean age at the time of surgery was 51.2 ± 17.9 years in the NSAID prescription cohort compared with 56.8 ± 17.8 years in the non-NSAID prescription cohort (*P* < 0.001). Female patients represented 65.4% of the NSAID cohort and 70.8% of the non-NSAID prescription cohort (*P* < 0.001). The mean body mass index (BMI) was 27.8 ± 6.56 kg/m² in the NSAID prescription cohort and 27.9 ± 6.62 kg/m² in the non-NSAID prescription cohort (*P* = 0.0716) (Table 1).

**Table 1:** Baseline demographic and clinical characteristics of patients undergoing DRF surgery with (*n* = 32,037) and without (*n* = 44,876) perioperative NSAID use, before propensity score matching

Category	Variable	NSAID Value	NSAID (%)	Non-NSAID value	Non-NSAID %	*P*-Value
Demographics	Age at index	51.2 ± 17.9	100	56.8 ± 17.8	100	< 0.0001
	White	25,230	78.753	35,963	80.139	< 0.0001
	Female	20,942	65.368	31,789	70.837	< 0.0001
	Hispanic or Latino	3657	11.415	3541	7.891	< 0.0001
	Black or African American	2472	7.716	3112	6.935	< 0.0001
	BMI	27.8 ± 6.56	73.231	27.9 ± 6.62	71.464	0.0716
Medications	Opioid analgesics	25,751	80.379	34,838	77.632	< 0.0001
	Glucocorticoids	13,025	40.656	16,660	37.125	< 0.0001
	Bisphosphonates	1132	3.533	2305	5.136	< 0.0001
Comorbidities	Hyperlipidemia	5726	17.873	10,620	23.665	< 0.0001
	Nicotine dependence	5275	16.465	6452	14.377	< 0.0001
	Vitamin D deficiency	3271	10.210	5565	12.401	< 0.0001
	Osteoporosis without fracture	2734	8.534	5390	12.011	< 0.0001
	Diabetes mellitus	2599	8.112	4802	10.701	< 0.0001
	Other anemias	2497	7.794	4676	10.420	< 0.0001
	COPD	1406	4.389	2798	6.235	< 0.0001
	Chronic kidney disease	822	2.566	2448	5.455	< 0.0001
	Alcohol dependence	764	2.385	1023	2.280	0.3400
	Heart failure	712	2.222	1895	4.223	< 0.0001
	Malnutrition	484	1.511	972	2.166	< 0.0001
	Rheumatoid arthritis	455	1.420	845	1.883	< 0.0001
	Osteoporosis with fracture	382	1.192	694	1.546	< 0.0001

Data are presented as mean ± standard deviation for continuous variables or number (percentage) for categorical variables. NSAID = nonsteroidal anti-inflammatory drug; BMI = body mass index. Categorical variables were compared using chi-square tests, and continuous variables were compared using independent t-tests. P-values < 0.05 were considered statistically significant.

After 1:1 propensity score matching, each cohort included 30,760 patients, and balance across demographic, clinical, and medication variables markedly improved. The matched NSAID prescription and non-NSAID prescription cohorts had nearly identical distributions of age, sex, and BMI. Comorbidities and medication use were similarly well balanced between groups after matching (Table 2).

**Table 2:** Baseline demographic and clinical characteristics of patients undergoing DRF surgery with (*n* = 30,760) and without (*n* = 30,760) perioperative NSAID use, after propensity score matching

Category	Variable	NSAID value	NSAID %	Non-NSAID value	Non-NSAID %	*P*-value
Demographics	Age at index	52.1 ± 17.5	100	52.0 ± 18.0	100	0.4432
	White	24,325	79.080	24,405	79.340	0.4267
	Female	20,439	66.447	20,453	66.492	0.9048
	Hispanic or Latino	3,178	10.332	3068	9.974	0.1420
	Black or African American	2,352	7.646	2261	7.350	0.1636
	BMI	27.8 ± 6.54	73.001	27.9 ± 6.61	73.618	0.2544
Medications	Opioid analgesics	24,578	79.902	24,389	79.288	0.0587
	Glucocorticoids	12,150	39.499	11,666	37.926	< 0.0001
	Bisphosphonates	1,132	3.680	1048	3.407	0.0670
Diagnoses	Hyperlipidemia	5,700	18.531	5435	17.669	0.0055
	Nicotine dependence	4,962	16.131	4745	15.426	0.0164
	Vitamin D deficiency	3,230	10.501	3057	9.938	0.0213
	Osteoporosis without fracture	2,733	8.885	2639	8.579	0.1794
	Diabetes mellitus	2,579	8.384	2360	7.672	0.0012
	Other anemias	2,463	8.007	2306	7.497	0.0179
	COPD	1,404	4.564	1264	4.109	0.0056
	Chronic kidney disease	822	2.672	732	2.380	0.0208
	Alcohol dependence	735	2.389	670	2.178	0.0794
	Heart failure	711	2.311	649	2.110	0.0891
	Malnutrition	480	1.560	437	1.421	0.1525
	Rheumatoid arthritis	455	1.479	422	1.372	0.2617
	Osteoporosis with fracture	379	1.232	344	1.118	0.1904

Data are presented as mean ± standard deviation for continuous variables or number (percentage) for categorical variables. NSAID = nonsteroidal anti-inflammatory drug; BMI = body mass index. Categorical variables were compared using chi-square tests, and continuous variables were compared using independent t-tests. P-values < 0.05 were considered statistically significant

### Surgical complications

Within 1 year of surgery, patients in the NSAID prescription cohort demonstrated higher rates of surgical complications. In the unmatched analysis, the incidence of the primary outcome of malunion/nonunion surgery was 0.66% (212/32,037) in the NSAID prescription cohort compared with 0.51% (227/44,876) in the non-NSAID prescription cohort, with an absolute risk difference of 0.16% (HR 1.33, 95% CI 1.10–1.60, *P* = 0.003). After propensity score matching, the incidence of malunion/nonunion surgery was 0.65% (201/30,760) in the NSAID prescription cohort compared with 0.55% (168/30,760) in the non-NSAID prescription cohort, with an absolute risk difference of 0.11% (HR 1.20, 95% CI 0.98–1.47, *P* = 0.083) (Table 3).

**Table 3:** Clinical outcomes in propensity score-matched cohorts of patients undergoing distal radius fracture surgery with (*n* = 30,760) and without (*n* = 30,760) perioperative NSAID use

Outcome	NSAID n (%)	Non-NSAID n (%)	Risk diff (95% CI)	Measure (OR, 95% CI)	p
*90-day medical outcomes*					
MI	65 (0.211%)	67 (0.218%)	-0.007% (-0.080%, 0.067%)	OR 0.970 (0.689–1.365)	0.8617
Gastrointestinal bleeding	57 (0.185%)	46 (0.150%)	0.036% (-0.029%, 0.100%)	OR 1.240 (0.840–1.829)	0.2780
Acute kidney injury	228 (0.741%)	225 (0.731%)	0.010% (-0.125%, 0.145%)	OR 1.013 (0.842–1.219)	0.8875
ED visit	2016 (6.554%)	1741 (5.660%)	0.894% (0.516%, 1.272%)	OR 1.169 (1.094–1.249)	< 0.0001
Readmission	1111 (3.612%)	1052 (3.420%)	0.192% (-0.099%, 0.483%)	OR 1.058 (0.971–1.153)	0.1965
*1-year surgical outcomes*					
Malunion / Nonunion surgery	201 (0.653%)	168 (0.546%)	0.107% (-0.015%, 0.229%)	HR 1.198 (0.976–1.471)	0.0831
Infection	415 (1.349%)	285 (0.927%)	0.423% (0.255%, 0.590%)	HR 1.460 (1.256–1.698)	< 0.0001
Revision surgery	658 (2.139%)	559 (1.817%)	0.322% (0.102%, 0.542%)	HR 1.182 (1.056–1.323)	0.0036

Data are presented as number (percentage), with risk differences (Risk Diff) and 95% confidence intervals (CI). NSAID = nonsteroidal anti-inflammatory drug; OR = odds ratio; HR = hazard ratio; MI = myocardial infarction; ED = emergency department.

Postoperative infection rates were higher in the NSAID prescription cohort. The incidence was 1.35% (415/30,760) in the NSAID prescription cohort compared with 0.93% (285/30,760) in the non-NSAID prescription cohort, with an absolute risk difference of 0.42% (HR 1.46, 95% CI 1.26–1.70, *P* < 0.001) (Table 3).

The incidence of revision surgery was 2.14% (658/30,760) in the NSAID prescription cohort compared with 1.82% (559/30,760) in the non-NSAID prescription cohort, with an absolute risk difference of 0.32% (HR 1.18, 95% CI 1.06–1.32, *P* = 0.004) (Table 3).

### Medical complications

Within 90 days of surgery, the rates of MI, GIB, and AKI did not differ significantly between cohorts (Table 3). Patients in the NSAID prescription cohort had a significantly higher rate of ED visits. The incidence was 6.55% in the NSAID prescription cohort compared with 5.66% in the non-NSAID prescription cohort, with an absolute risk difference of 0.89% (OR 1.17, 95% CI 1.09–1.25, *P* < 0.001). The rate of inpatient readmission was 3.61% in the NSAID prescription cohort compared with 3.42% in the non-NSAID prescription cohort, with an absolute risk difference of 0.19% (OR 1.06, 95% CI 0.97–1.15, *P* = 0.197) (Table 3).

### Sensitivity analyses

After excluding patients who received a COX-2 selective inhibitor prescription (25,095 matched pairs), the primary outcome of malunion/nonunion surgery remained non-significant (0.68% vs. 0.57%, HR 1.19, 95% CI 0.95–1.48, *P* = 0.129). Postoperative infection remained significantly higher in the NSAID cohort (1.06% vs. 0.67%, HR 1.59, 95% CI, 1.31–1.93, *P* < 0.0001), as did revision surgery (2.33% vs. 1.91%, HR 1.23, 95% CI 1.09–1.39, *P* = 0.0008). The rate of ED visits was higher in the NSAID cohort (15.42% vs. 14.47%, HR 1.08, 95% CI 1.03–1.13, log-rank *P* = 0.0006). When the exposure window was narrowed to prescriptions within 1 week of surgery (27,468 matched pairs), results were similar: malunion/nonunion surgery showed no significant difference (0.65% vs. 0.60%, HR 1.09, 95% CI 0.88–1.35, log-rank *P* = 0.41), while infection (1.33% vs. 1.02%, HR 1.32, 95% CI 1.13–1.55, *P* = 0.0014) and revision surgery (2.21% vs. 1.93%, HR 1.16, 95% CI 1.03–1.30, *P* = 0.014) remained significantly elevated. ED visits were modestly higher in the NSAID cohort but did not reach significance in this restricted window (14.65% vs. 14.24%, HR 1.04, 95% CI 1.00-1.09, log-rank *P* = 0.068). Full results of both sensitivity analyses are presented in Supplementary Tables 4 and 5.

## Discussion

This study evaluated postoperative complications associated with perioperative NSAID use following DRF surgery in a large, multi-institutional database. After propensity score matching, we identified statistically significant increases in postoperative infections, revision surgery, and ED visits among patients who received NSAID prescriptions. However, the absolute risk differences were modest, with increases of less than 0.5% for most outcomes. No significant differences were observed in major medical complications including MI, GIB, or AKI.

The infection risk was associated with 0.42% increase, translating to approximately 4 additional infections per 1,000 patients treated with NSAID prescriptions. Similarly, revision surgery rates increased by approximately 3 additional revisions per 1,000 patients. The primary outcome of malunion/nonunion surgery showed a non-significant increase of 0.11% after matching, despite reaching statistical significance in the unmatched analysis. These modest absolute risk increases must be interpreted in the context of limited effective alternatives for perioperative pain management following DRF surgery.

In our matched cohort analysis, we observed no significant difference in malunion/nonunion surgery rates (0.65% vs. 0.55%, HR 1.20, *P* = 0.083), consistent with multiple randomized controlled trials that found no adverse effects of NSAIDs on fracture unions of DRFs [12, 18, 19]. Aliuskevicius et al. conducted a triple-blinded randomized trial of 95 patients with displaced DRF treated with external fixation and found no differences in bone mineral density, histomorphometric characteristics of fracture callus, or bone turnover biomarkers between patients receiving ibuprofen (either 3 or 7 days) and placebo at 6 weeks, 3 months, and 1 year follow-up [18]. The statistically significant association observed in our unmatched analysis likely reflected baseline differences between patients prescribed NSAIDs and those who were not, rather than a true effect of NSAIDs on fracture healing. This null finding was robust to sensitivity analyses restricting the exposure definition to nonselective NSAIDs and to a shorter 1-week postoperative window, arguing against the possibility that heterogeneity in NSAID type or duration of exposure obscured a true effect on fracture union.

The absolute increase in postoperative infection rates (0.42%, from 0.93 to 1.35%) represents our most clinically notable finding, though few prior studies have specifically examined this outcome following DRF surgery. Jeffcoach et al. found a 2.17-fold increased risk of combined infectious and bone healing complications in 1901 patients receiving NSAIDs after long-bone fractures [24]. The biological mechanism may involve impaired inflammatory responses essential for wound healing and bacterial clearance, as NSAIDs inhibit prostaglandin synthesis critical to both inflammation and immune function [4]. Notably, the infection association was of similar or greater magnitude when the exposure definition was restricted to nonselective NSAIDs (HR 1.59) or to a 1-week postoperative window (HR 1.32), suggesting that this finding was not driven by inclusion of COX-2 selective agents or by prolonged NSAID exposure. However, our database did not capture perioperative antibiotic prophylaxis regimen, wound classification, or operative duration, all of which are established risk factors for surgical site infection [25, 26]. We cannot exclude the possibility that differences in antibiotic stewardship or surgical factors, rather than NSAID use itself, contributed to the observed infection risk.

The 0.89% absolute increase in ED visits within 90 days may reflect several factors. Inadequate pain control may necessitate emergency evaluation in some patients. However, this association may also represent residual confounding despite propensity score matching. Patients prescribed NSAIDs may have had greater baseline comorbidity burden, more complex fracture patterns, or other unmeasured clinical characteristics that independently increase healthcare utilization. Additionally, increased surgical complications, particularly infections and wound issues, may have prompted emergency visits for concerning postoperative symptoms.

The absence of significant differences in MI, GIB, and AKI is reassuring. The low absolute incidence of these events (MI 0.21–0.22%, GIB 0.15–0.19%, AKI 0.73–0.74%) and the short exposure duration in our study (perioperative period only) likely explain these findings. Previous large-scale studies have demonstrated dose- and duration-dependent relationships between NSAID exposure and cardiovascular and gastrointestinal risks [27, 28]. Our findings should not be interpreted as definitive evidence of safety in high-risk populations, but they suggest that short-term perioperative NSAID use does not substantially increase these serious complications in typical patients undergoing DRF surgery.

This study has several limitations inherent to retrospective database analyses. Accurate diagnostic and procedural coding cannot be verified, and misclassification may affect results. We could not assess fracture characteristics, surgical techniques, or fixation methods that influence outcomes. Although propensity score matching controlled for measured confounders, unmeasured factors such as surgeon experience, institutional protocols, patient preferences, fracture severity, and comorbidity burden may influence both NSAID prescription patterns and outcomes. The database does not capture over-the-counter NSAID use, potentially misclassifying some patients in the control group. Also, we did not evaluate alternative pain management strategies, limiting our ability to make comparative effectiveness recommendations. Our primary outcome, malunion/nonunion surgery, relies on procedural coding and therefore reflects a decision to reoperate rather than a direct measure of bone healing; this outcome may be influenced by surveillance bias or differential thresholds for reintervention between cohorts. A diagnosis-code-based definition of nonunion was not used because the applicable codes are fragmented across fracture type, laterality, and encounter type, precluding a single reliable case definition within this database. Similarly, we were unable to capture antibiotic prophylaxis regimen, wound classification, or operative duration, which limits our ability to fully account for confounding in the infection outcome, and emergency department visits could not be stratified by reason for visit.

Despite statistically significant associations, the absolute increases in complications associated with perioperative NSAID use following DRF surgery are modest. The clinical significance of these small absolute risk differences, less than 1% for all outcomes, must be weighed against the demonstrated effectiveness of NSAIDs for pain control and the limited alternative options for opioid-sparing analgesia [1]. This study did not evaluate analgesic efficacy, opioid consumption, or patient-reported pain outcomes, and therefore cannot directly support a comparative recommendation regarding perioperative analgesic choice. Rather, these findings indicate that the small absolute risks of infection and revision surgery associated with perioperative NSAID use should be weighed individually against a patient’s specific risk factors and the anticipated benefits of NSAID-based multimodal analgesia, with the treating surgeon making this determination on a case-by-case basis.

## Supplementary Information

Below is the link to the electronic supplementary material.

Supplementary Material 1.

Supplementary Material 2.

## Data Availability

The data that support the findings of this study were obtained from the TriNetX Research Network. Restrictions apply to the availability of these data, which were used under institutional license for the current study and are therefore not publicly available. Data may be available from TriNetX (https://trinetx.com/) upon reasonable request and with appropriate institutional access and data use agreements.

## References

[1] Sinatra RS, Torres J, Bustos AM (2002) Pain management after major orthopaedic surgery: current strategies and new concepts. JAAOS J Am Acad Orthop Surg 10:117–129 10.5435/00124635-200203000-00007 11929206

[2] Joshi GP, Beck DE, Emerson RH, Halaszynski TM, Jahr JS, Lipman AG et al (2014) Defining new directions for more effective management of surgical pain in the United States: highlights of the inaugural surgical pain congress™. Am Surg 80:219–228 24666860

[3] Chen X, Chu Q, Peng Y, Chen Y, Kaye AD, Liu H et al (2025) Clinical practice guidelines for postoperative pain management in adults (2024 edition). J Anesth Transl Med 4:161–185 10.1016/j.jatmed.2025.09.001 PMC13001796 41929376

[4] Ryan PM, Scherry H, Pierson R, Wilson CD, Probe RA (2024) NSAID use in orthopedic surgery: A review of current evidence and clinical practice guidelines. J Orthop Res 42:707–716 10.1002/jor.25791 38273720

[5] Gerstenfeld LC, Al-Ghawas M, Alkhiary YM, Cullinane DM, Krall EA, Fitch JL et al (2007) Selective and nonselective cyclooxygenase-2 inhibitors and experimental fracture-healing. Reversibility of effects after short-term treatment. J Bone Joint Surg Am 89:114–125 10.2106/JBJS.F.00495 17200318

[6] Simon AM, Manigrasso MB, O’Connor JP (2002) Cyclo-oxygenase 2 function is essential for bone fracture healing. J Bone Min Res 17:963–976 10.1359/jbmr.2002.17.6.963 12054171

[7] Bergenstock M, Min W, Simon AM, Sabatino C, O’Connor JP (2005) A comparison between the effects of acetaminophen and celecoxib on bone fracture healing in rats. J Orthop Trauma 19:717–723 10.1097/01.bot.0000184144.98071.5d 16314720

[8] Simon AM, O’Connor JP (2007) Dose and time-dependent effects of cyclooxygenase-2 inhibition on fracture-healing. J Bone Joint Surg Am 89:500–511 10.2106/JBJS.F.00127 17332098

[9] Kim H, Kim DH, Kim DM, Kholinne E, Lee ES, Alzahrani WM et al (2021) Do Nonsteroidal anti-inflammatory or COX-2 inhibitor drugs increase the nonunion or delayed union rates after fracture surgery? A propensity-score-matched study. J Bone Joint Surg 103:1402–1410 10.2106/JBJS.20.01663 34101675

[10] Wheatley BM, Nappo KE, Christensen DL, Holman AM, Brooks DI, Potter BK (2019) Effect of NSAIDs on bone healing rates: a meta-analysis. J Am Acad Orthop Surg 27:e330–e6 10.5435/JAAOS-D-17-00727 30260913

[11] Marquez-Lara A, Hutchinson ID, Nunez F Jr., Smith TL, Miller AN (2016) Nonsteroidal anti-inflammatory drugs and bone-healing: a systematic review of research quality. JBJS Rev. 4 10.2106/JBJS.RVW.O.00055 27500434

[12] Aliuskevicius M, Østgaard SE, Vestergaard P, Rasmussen S (2021) The influence of ibuprofen on the healing of nonsurgically treated colles’ fractures. Orthopedics 44:105–110 10.3928/01477447-20201216-04 33373464

[13] Thomas AP, Brooks S (1986) The use of an oral prostaglandin inhibitor following splintage in fractures of the distal radius—a prospective trial. Injury 17:179–181 10.1016/0020-1383(86)90328-1 3546124

[14] Zura R, Xiong Z, Einhorn T, Watson JT, Ostrum RF, Prayson MJ et al (2016) Epidemiology of fracture nonunion in 18 human bones. JAMA Surg. 151 10.1001/jamasurg.2016.2775 27603155

[15] Court-Brown CM, Caesar B (2006) Epidemiology of adult fractures: a review. Injury 37:691–697 10.1016/j.injury.2006.04.130 16814787

[16] Barrington R (1980) Single-blind study of diflunisal versus mefenamic acid in the treatment of pain after Colles’ fracture. Curr Med Res Opin 6:630–633 10.1185/03007998009109500 7408529

[17] Adolphson P, Abbaszadegan H, Jonsson U, Dalén N, Sjöberg HE, Kalén S (1993) No effects of piroxicam on osteopenia and recovery after Colles’ fracture. A randomized, double-blind, placebo-controlled, prospective trial. Arch Orthop Trauma Surg 112:127–130 10.1007/BF00449987 8323840

[18] Aliuskevicius M, Østgaard SE, Hauge EM, Vestergaard P, Rasmussen S (2020) Influence of ibuprofen on bone healing after colles’ fracture: a randomized controlled clinical trial. J Orthop Res 38:545–554 10.1002/jor.24498 31646668

[19] Aliuskevicius M, Østgaard SE, Rasmussen S (2019) No influence of ibuprofen on bone healing after Colles’ fracture—a randomized controlled clinical trial. Injury 50:1309–1317 10.1016/j.injury.2019.06.011 31186121

[20] Luo P, Lou J, Yang S (2018) Pain management during rehabilitation after distal radius fracture stabilized with volar locking plate: a prospective cohort study. Biomed Res Int 2018:5786089 10.1155/2018/5786089 PMC6241235 30519581

[21] Chang M, Kasper A, Chung J, Wright J, Pennington M, Ilyas AM (2025) The effect of nonsteroidal anti-inflammatory drugs on union rates following operative repair of distal radius fractures. J Hand Surg Glob Online 7:100720 10.1016/j.jhsg.2025.03.001 PMC12148464 40497268

[22] Wang Z, Bhattacharyya T (2015) Trends of non-union and prescriptions for non-steroidal anti-inflammatory drugs in the United States, 1993–2012. Acta Orthop 86:632–637 10.3109/17453674.2015.1028860 PMC4564789 25761793

[23] McDonald E, Winters B, Nicholson K, Shakked R, Raikin S, Pedowitz DI et al (2018) Effect of postoperative ketorolac administration on bone healing in ankle fracture surgery. Foot Ankle Int 39:1135–1140 10.1177/1071100718782489 29972028

[24] Jeffcoach DR, Sams VG, Lawson CM, Enderson BL, Smith ST, Kline H et al (2014) Nonsteroidal anti-inflammatory drugs’ impact on nonunion and infection rates in long-bone fractures. J Trauma Acute Care Surg 76:779–783 10.1097/TA.0b013e3182aafe0d 24553548

[25] Yang Z, Meng H, Li J, Du P, Lv H, Zhao K et al (2025) Association between the generation of cephalosporins for perioperative prophylaxis and postoperative surgical site infections in open fractures: a prospective cohort study. Int J Surg 111:3810–3820 10.1097/JS9.0000000000002371 PMC12165470 40202921

[26] Yang Z, Wang Y, Wang Z, Li J, Du P, Meng H et al (2025) Duration of surgical antibiotic prophylaxis and surgical site infection in orthopaedic surgery: a prospective cohort study. Int J Surg 111:492–501 10.1097/JS9.0000000000001881 PMC11745748 39017708

[27] Langman MJ, Weil J, Wainwright P, Lawson DH, Rawlins MD, Logan RF et al (1994) Risks of bleeding peptic ulcer associated with individual non-steroidal anti-inflammatory drugs. Lancet 343:1075–1078 10.1016/s0140-6736(94)90185-6 7909103

[28] Garcia Rodriguez LA, Tacconelli S, Patrignani P (2008) Role of dose potency in the prediction of risk of myocardial infarction associated with nonsteroidal anti-inflammatory drugs in the general population. J Am Coll Cardiol 52:1628–1636 10.1016/j.jacc.2008.08.041 18992652

