# Supplementary material for: Perioperative NSAID use and postoperative outcomes after distal radius fracture surgery: a propensity-matched cohort study
Source: Eur J Orthop Surg Traumatol. 2026 Sep 30;36(1):397. doi: 10.1007/s00590-026-04980-y (PMC13627179; doi:10.1007/s00590-026-04980-y)
Supplement: Supplementary file 1 [file 590_2026_4980_MOESM1_ESM.docx]

**Supplementary Table 1. ICD-10, CPT, and RxNorm Codes Used for Cohort Identification, Propensity Score Matching, and Outcome Assessment**

| **Code Type** | **Description** | **Code** |
| --- | --- | --- |
|  | COHORT 1: NSAID GROUP |  |
| CPT | Open treatment of distal radial extra-articular fracture or epiphyseal separation, with internal fixation | 25607 |
| CPT | Open treatment of distal radial intra-articular fracture or epiphyseal separation, with internal fixation of 2 fragments | 25608 |
| CPT | Open treatment of distal radial intra-articular fracture or epiphyseal separation, with internal fixation of 3 or more fragments | 25609 |
|  | AND any of the following NSAID medications: |  |
| RxNorm | Etodolac | 24605 |
| VA | Non-steroidal anti-inflammatory analgesics | CN104 |
| RxNorm | Ibuprofen | 5640 |
| RxNorm | Naproxen | 7258 |
| RxNorm | Diclofenac | 3355 |
| RxNorm | Ketorolac | 35827 |
| RxNorm | Nabumetone | 31448 |
| RxNorm | Piroxicam | 8356 |
| RxNorm | Sulindac | 10237 |
| RxNorm | Oxaprozin | 32613 |
| RxNorm | Celecoxib | 140587 |
| RxNorm | Indomethacin | 5781 |
|  |  |  |
|  | COHORT 2: NON-NSAID GROUP |  |
| CPT | Open treatment of distal radial extra-articular fracture or epiphyseal separation, with internal fixation | 25607 |
| CPT | Open treatment of distal radial intra-articular fracture or epiphyseal separation, with internal fixation of 2 fragments | 25608 |
| CPT | Open treatment of distal radial intra-articular fracture or epiphyseal separation, with internal fixation of 3 or more fragments | 25609 |
|  | CANNOT have any of the following: |  |
| VA | Non-steroidal anti-inflammatory analgesics | CN164 |
| ATC | Coxibs | M01AH |
| RxNorm | Etodolac | 24605 |
| RxNorm | Piroxicam | 8356 |
| RxNorm | Ketorolac | 35827 |
| RxNorm | Celecoxib | 140587 |
| RxNorm | Sulindac | 10237 |
| RxNorm | Indomethacin | 5781 |
| RxNorm | Oxaprozin | 32613 |
| RxNorm | Nabumetone | 31448 |
| RxNorm | Diclofenac | 3355 |
| RxNorm | Naproxen | 7258 |
| RxNorm | Ibuprofen | 5640 |
|  |  |  |
|  | PROPENSITY SCORE MATCHING COVARIATES |  |
| Demographic | Age at index | Continuous |
| Demographic | Female | — |
| Demographic | Hispanic or Latino | 2135-2 |
| Demographic | White | 2106-3 |
| Demographic | Black or African American | 2054-5 |
| TNX Curated | Body mass index (BMI) categories | 9083 |
| ATC | Bisphosphonates | M05BA |
| VAC | Opioid analgesics | N101 |
| ICD-10-CM | Diabetes mellitus | E08-E13 |
| ICD-10-CM | Osteoporosis without current pathological fracture | M81 |
| ICD-10-CM | Osteoporosis with current pathological fracture | M80 |
| ICD-10-CM | Nicotine dependence | F17 |
| ICD-10-CM | Alcohol dependence | F10.2 |
| ICD-10-CM | Heart failure | I50 |
| ICD-10-CM | Other anemias | D64 |
| ICD-10-CM | Other chronic obstructive pulmonary disease | J44 |
| ICD-10-CM | Hyperlipidemia, unspecified | E78.5 |
| ICD-10-CM | Vitamin D deficiency | E55 |
| ICD-10-CM | Chronic kidney disease | N18 |
| ICD-10-CM | Malnutrition | E40-E46 |
| ATC | Glucocorticoids | H02AB |
| ICD-10-CM | Rheumatoid arthritis, unspecified | M06.9 |
|  |  |  |
|  | OUTCOMES |  |
|  | Postoperative Infection (1 year) |  |
| ICD-10-CM | Infection following a procedure | T81.4 |
| ICD-10-CM | Infection and inflammatory reaction due to internal fixation device of arm | T84.61 |
| ICD-10-CM | Infection and inflammatory reaction due to internal fixation device of unspecified site, initial encounter | T84.60XA |
|  |  |  |
|  | Malunion/Nonunion Surgery (1 year) |  |
| CPT | Repair of nonunion or malunion, radius OR ulna; without graft (eg, compression technique) | 25400 |
| CPT | Repair of nonunion or malunion, radius OR ulna; with autograft (includes obtaining graft) | 25405 |
| CPT | Repair of nonunion or malunion, radius AND ulna; without graft (eg, compression technique) | 25415 |
| CPT | Repair of nonunion or malunion, radius AND ulna; with autograft (includes obtaining graft) | 25420 |
| CPT | Repair of nonunion or malunion, radius OR ulna | 1004522 |
| CPT | Repair of nonunion or malunion, radius AND ulna | 1004525 |
|  |  |  |
|  | Revision Surgery (1 year) |  |
| CPT | Repair, Revision, and/or Reconstruction Procedures on the Forearm and Wrist | 1004481 |
| CPT | Repair of nonunion or malunion, radius OR ulna; without graft (eg, compression technique) | 25400 |
| CPT | Repair of nonunion or malunion, radius OR ulna; with autograft (includes obtaining graft) | 25405 |
| CPT | Repair of nonunion or malunion, radius AND ulna; without graft (eg, compression technique) | 25415 |
| CPT | Repair of nonunion or malunion, radius AND ulna; with autograft (includes obtaining graft) | 25420 |
| CPT | Repair of defect with autograft; radius OR ulna | 25425 |
| CPT | Repair of defect with autograft; radius AND ulna | 25426 |
| CPT | Repair of nonunion or malunion, radius OR ulna | 1004522 |
| CPT | Repair of nonunion or malunion, radius AND ulna | 1004525 |
|  |  |  |
|  | Emergency Department Visit (90 days) |  |
| CPT | Emergency department visit, may not require physician presence | 99281 |
| CPT | Emergency department visit, straightforward medical decision making | 99282 |
| CPT | Emergency department visit, low level of medical decision making | 99283 |
| CPT | Emergency department visit, moderate level of medical decision making | 99284 |
| CPT | Emergency department visit, high level of medical decision making | 99285 |
|  |  |  |
|  | Inpatient Readmission (90 days) |  |
| CPT | Initial hospital care, straightforward or low level decision making (40 min) | 99221 |
| CPT | Initial hospital care, moderate level decision making (55 min) | 99222 |
| CPT | Initial hospital care, high level decision making (75 min) | 99223 |
| CPT | Same-day admission and discharge, straightforward or low level (45 min) | 99234 |
| CPT | Same-day admission and discharge, moderate level (70 min) | 99235 |
| CPT | Same-day admission and discharge, high level (85 min) | 99236 |
|  |  |  |
|  | Acute Kidney Injury (90 days) |  |
| ICD-10-CM | Acute kidney failure | N17 |
|  |  |  |
|  | Gastrointestinal Bleeding (90 days) |  |
| ICD-10-CM | Gastrointestinal hemorrhage, unspecified | K92.2 |
| ICD-10-CM | Gastritis, unspecified, with bleeding | K29.71 |
|  |  |  |
|  | Myocardial Infarction (90 days) |  |
| ICD-10-CM | Acute myocardial infarction | I21 |
