# Supplementary material for: Perioperative NSAID use and postoperative outcomes after distal radius fracture surgery: a propensity-matched cohort study
Source: Eur J Orthop Surg Traumatol. 2026 Sep 30;36(1):397. doi: 10.1007/s00590-026-04980-y (PMC13627179; doi:10.1007/s00590-026-04980-y)
Supplement: Supplementary file 2 [file 590_2026_4980_MOESM2_ESM.docx]

**Supplementary Table 4**

Sensitivity Analysis Excluding COX-2 Selective Inhibitor Prescriptions: Clinical Outcomes in Propensity Score-Matched Cohorts of Patients Undergoing Distal Radius Fracture Surgery With (n=25,095) and Without (n=25,095) Perioperative Nonselective NSAID Use

| **90-Day Medical Outcomes** | | | | | |
| --- | --- | --- | --- | --- | --- |
| **Outcome** | **NSAID n (%)** | **non-NSAID n (%)** | **Risk Diff (95% CI)** | **Measure (HR/OR, 95% CI)** | **p** |
| MI | 55 (0.219%) | 70 (0.279%) | -0.060% (-0.147%, 0.027%) | OR 0.786 (0.552-1.118) | 0.1792 |
| Gastrointestinal bleeding | 53 (0.211%) | 44 (0.175%) | 0.036% (-0.041%, 0.113%) | OR 1.205 (0.808-1.798) | 0.3604 |
| Acute kidney injury | 212 (0.845%) | 196 (0.781%) | 0.064% (-0.093%, 0.221%) | OR 1.082 (0.891-1.315) | 0.4264 |
| ED visit | 3,869 (15.417%) | 3,631 (14.469%) | 0.948% (0.325%, 1.572%) | OR 1.077 (1.026-1.132) | 0.0029 |
| Readmission | 895 (3.566%) | 867 (3.455%) | 0.112% (-0.210%, 0.434%) | OR 1.033 (0.940-1.137) | 0.4971 |
|  | | | | | |
| **1-Year Surgical Outcomes** | | | | | |
| **Outcome** | **NSAID n (%)** | **non-NSAID n (%)** | **Risk Diff (95% CI)** | **Measure (HR, 95% CI)** | **p** |
| Malunion / Nonunion surgery | 170 (0.677%) | 144 (0.574%) | 0.104% (-0.034%, 0.242%) | HR 1.187 (0.951-1.482) | 0.1411 |
| Infection | 267 (1.064%) | 169 (0.673%) | 0.391% (0.228%, 0.553%) | HR 1.594 (1.314-1.932) | <0.0001 |
| Revision surgery | 585 (2.331%) | 480 (1.913%) | 0.418% (0.166%, 0.671%) | HR 1.229 (1.089-1.386) | 0.0011 |

*Data are presented as number (percentage), with risk differences (Risk Diff) and 95% confidence intervals (CI). NSAID = nonsteroidal anti-inflammatory drug; COX-2 = cyclooxygenase-2; OR = odds ratio; HR = hazard ratio; MI = myocardial infarction; ED = emergency department.*

**Supplementary Table 5**

Sensitivity Analysis Restricting NSAID Exposure to a 1-Week Postoperative Window: Clinical Outcomes in Propensity Score-Matched Cohorts of Patients Undergoing Distal Radius Fracture Surgery With (n=27,468) and Without (n=27,468) Perioperative NSAID Use

| **90-Day Medical Outcomes** | | | | | |
| --- | --- | --- | --- | --- | --- |
| **Outcome** | **NSAID n (%)** | **non-NSAID n (%)** | **Risk Diff (95% CI)** | **Measure (HR/OR, 95% CI)** | **p** |
| MI | 149 (0.542%) | 162 (0.590%) | -0.047% (-0.173%, 0.078%) | OR 0.919 (0.735-1.149) | 0.4598 |
| Gastrointestinal bleeding | 104 (0.379%) | 92 (0.335%) | 0.044% (-0.056%, 0.143%) | OR 1.131 (0.854-1.498) | 0.3905 |
| Acute kidney injury | 417 (1.518%) | 449 (1.635%) | -0.116% (-0.325%, 0.092%) | OR 0.928 (0.811-1.061) | 0.2730 |
| ED visit | 4,024 (14.65%) | 3,911 (14.238%) | 0.411% (-0.177%, 0.999%) | OR 1.034 (0.986-1.084) | 0.1702 |
| Readmission | 1,543 (5.617%) | 1,536 (5.592%) | 0.025% (-0.359%, 0.410%) | OR 1.005 (0.934-1.081) | 0.8967 |
|  | | | | | |
| **1-Year Surgical Outcomes** | | | | | |
| **Outcome** | **NSAID n (%)** | **non-NSAID n (%)** | **Risk Diff (95% CI)** | **Measure (HR, 95% CI)** | **p** |
| Malunion / Nonunion surgery | 178 (0.648%) | 164 (0.597%) | 0.051% (-0.081%, 0.183%) | HR 1.086 (0.878-1.343) | 0.4476 |
| Infection | 366 (1.332%) | 279 (1.016%) | 0.317% (0.137%, 0.497%) | HR 1.316 (1.125-1.539) | 0.0006 |
| Revision surgery | 607 (2.210%) | 530 (1.930%) | 0.280% (0.042%, 0.518%) | HR 1.149 (1.021-1.292) | 0.0210 |

*Data are presented as number (percentage), with risk differences (Risk Diff) and 95% confidence intervals (CI). NSAID exposure was defined as any NSAID prescription from 1 day before to 1 week after the index surgical procedure. NSAID = nonsteroidal anti-inflammatory drug; OR = odds ratio; HR = hazard ratio; MI = myocardial infarction; ED = emergency department.*
